# A 53-year-old man with a sclerosing rib lesion: A case report

**DOI:** 10.1097/MD.0000000000008692

**Published:** 2017-11-27

**Authors:** Shucai Bai, Huafeng Zhang, Zhijun Li, Dong Li, Hui Li

**Affiliations:** Department of Orthopedics, Tianjin Medical University General Hospital, Tianjin, China.

**Keywords:** chest, osteomyelitis, rib

## Abstract

**Rationale:**

Sclerosing osteomyelitis of Garré is a rare condition that occurs most commonly in tubular bones and the mandible. Its nontypical symptoms, low morbidity, and insidious process make its diagnosis difficult at an early stage. In this article, we reported a case of chronic sclerosing osteomyelitis which occurred in flat bone.

**Patient concerns:**

A 53-year-old man was diagnosed with rib sclerosing osteomyelitis of Garré who had an 8-year course of intermittent local pain and swelling, which radiated toward the left side of his chest wall. Chest computed tomography (CT) showed irregular sclerosis of the diaphysis of the 10th rib, with periosteal reaction and narrowing of the medullary cavity, and magnetic resonance imaging (MRI) showed T2 heterogeneous low-signal intensity over the 10th rib.

**Diagnoses:**

Based on the features of the clinical signs and radiography and biopsy of the lesion, diagnosis of rib sclerosing osteomyelitis of Garré was made.

**Interventions:**

The patient was treated with surgical excision of a 10-cm-long lesion after failed conservative treatment.

**Outcomes:**

Postoperatively, the patient achieved good functional recovery at the 10-year follow-up.

**Lessons:**

Rib sclerosing osteomyelitis of Garré is an unusual condition and represents a noninfective course in the rib with a low morbidity. The surgical management was successful in relieving the patient's symptom.

## 1 Introduction

Sclerosing osteomyelitis of Garré is a chronic disease characterized by peripheral and periosteal reactive bone formation, with an elusive course.^[1]^ It is often described as non-suppurative chronic sclerosing osteomyelitis or periostitis ossificans. The etiology of sclerosing osteomyelitis of Garré remains controversial and is believed to be a low viral infection or irritation.^[2,3]^ One study^[4]^ proposed it is related to the immune status of the host, whereas another study^[5]^ reported it to be a complication of management of surgical fixation of femur fracture. The disease primarily affects the mandible and the diaphysis of long bones, but it can also affect any bone (Table 1).^[1,6,7]^ Herein, we present a rare case of a patient with chronic chest pain and swelling which was ultimately diagnosed as sclerosing osteomyelitis of the rib. The patient was successfully treated with a complete surgical resection.

**Table 1:**
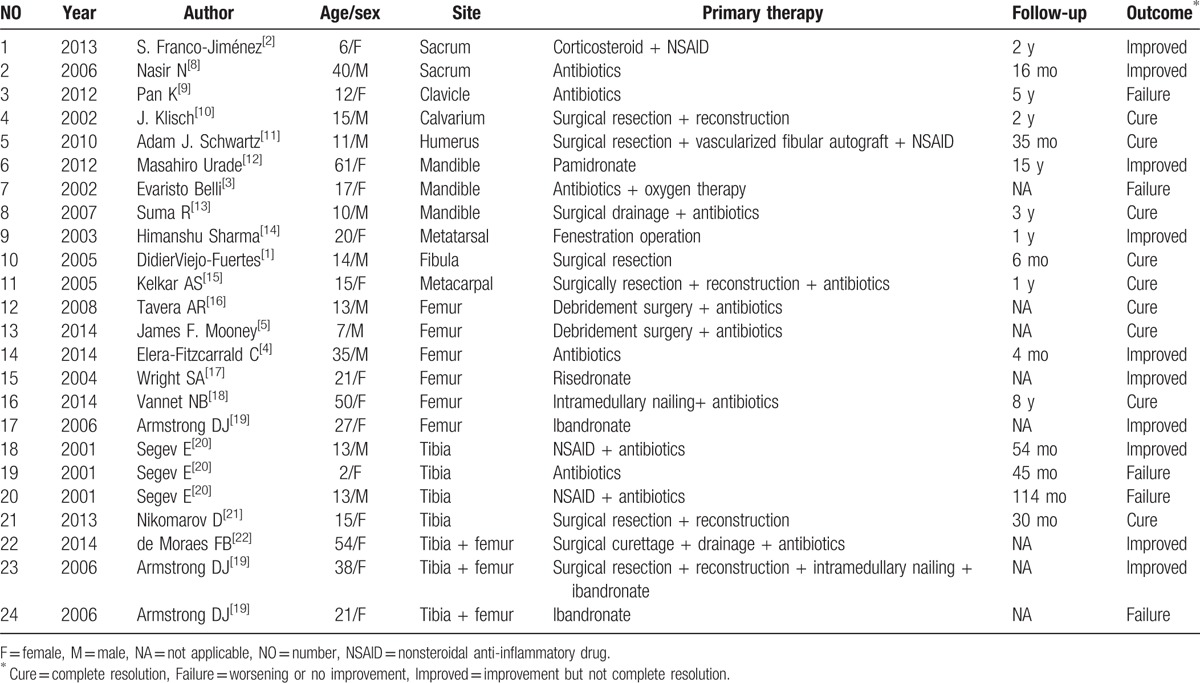
Published cases of sclerosing osteomyelitis of Garré since 2000 in the literature.

## 2 Case presentation

A 53-year-old man was referred to our department in August 2006 with an 8-year course of intermittent local pain and swelling, which radiated toward the left side of his chest. The patient had no history of previous infection, fever, and trauma. In addition, there was no evidence of any acne and pustule on his skin.

**Figure 1:**
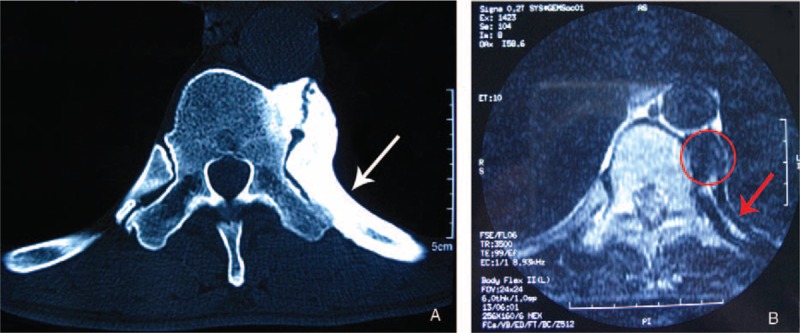
(A) Noncontrast axial chest computerized tomography (CT) scan showing irregular sclerosis of the diaphysis of the 10th rib (white arrow), with periosteal reaction and narrowing of the medullary cavity. (B) Axial T1-weighted MRI at the level of 10th thoracic vertebra showing heterogeneous low signal intensity over the 10th rib (red arrow). Sclerosing osteophytes can be seen on the left side of the 10th thoracic vertebra (circle).

On visiting our department in August 2006, the patient's physical examination showed localized tenderness and swelling over the 10th thoracic vertebra and 10th rib. He also had chronic limitation of movement in his spine. Preoperative chest computed tomography (CT) showed irregular sclerosis of the diaphysis of the 10th rib, with periosteal reaction and narrowing of the medullary cavity (Fig. 1A), and magnetic resonance imaging (MRI) showed heterogeneous low-signal intensity over the 10th rib (Fig. 1B). However, no bone sequestration was observed.

His blood and biochemical test results were normal. The erythrocyte sedimentation rate was 9 mm/h. In addition, the preoperative examination result of core needle biopsy showed no anaerobic organisms or tumor cells. Microscopic examination suggested chronic inflammatory changes with osteoblastic periosteal reaction. Based on the features of the clinical signs, radiography and biopsy of puncture specimen, the rib sclerosing osteomyelitis of Garré was initially diagnosed. The patient received conservative treatment. However, the symptoms persisted intermittently despite treatment with nonsteroidal anti-inflammatory drugs (NSAIDs) (oral ibuprofen 300 mg/12 h), and the intensity of the pain increased during next 3 months around the lesion of 10th thoracic vertebra and 10th rib.

**Figure 2:**
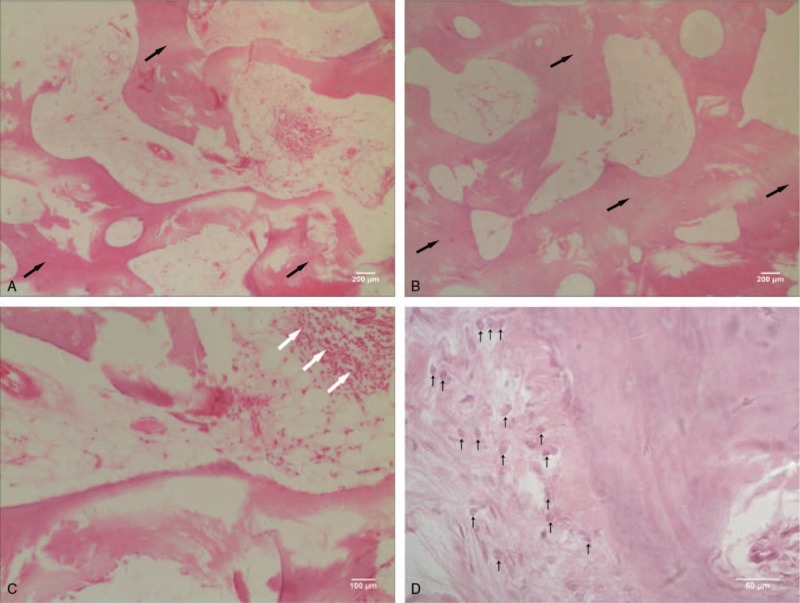
(A, B) The biopsy specimen (hematoxylin and eosin staining, 40× magnification) showing sclerosing bone formation with thickened irregular trabeculae (black arrow). (C) The biopsy specimen (hematoxylin and eosin staining, 100× magnification) showing an inflammatory infiltration of lymphocytes and plasma cells (white arrow area). (D) The biopsy specimen (hematoxylin and eosin staining, 400× magnification) showing sclerosing bone formation surrounded by fibrous stroma with osteoblasts (black arrow).

**Figure 3:**
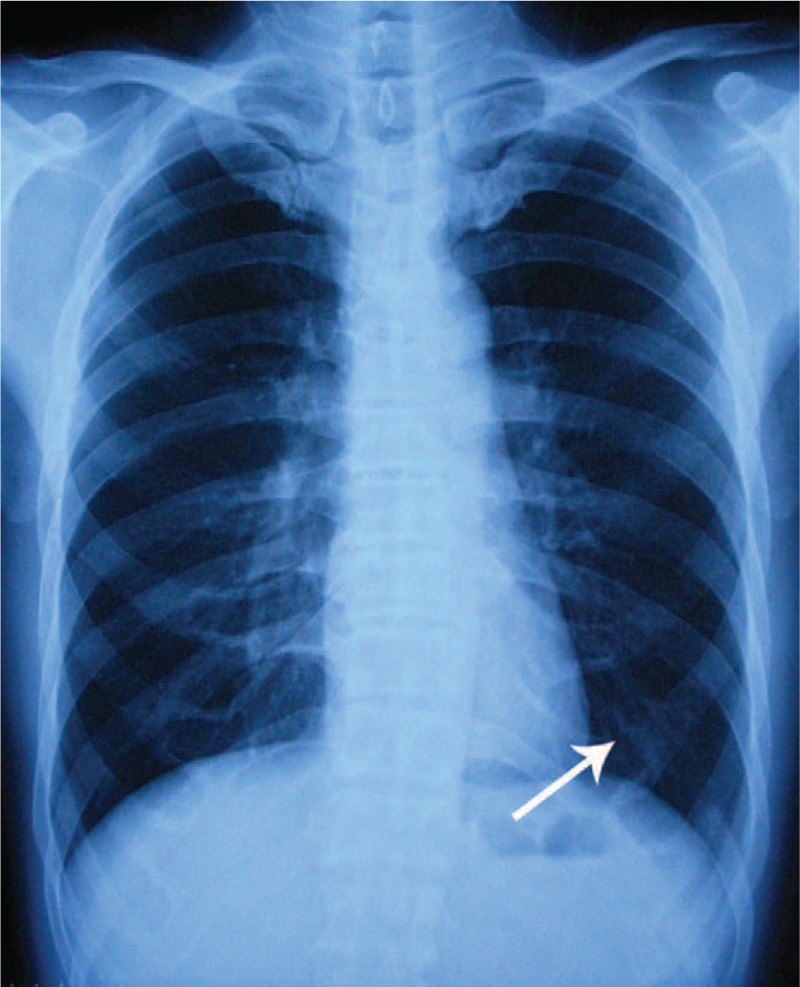
The chest radiograph showing the resected 10th rib and no recurrent lesion at the 10-y follow-up.

A skeletal surgery was performed for our patient, and no abscess or necrotic tissue was detected. We found a rib lesion with a diameter of 5 cm and the length of the sclerosing bone without the medullary cavity was 10 cm. The lesion was oval and slippery, and resembled ivory. Subsequently, the lesion of the 10th rib, 10th intercostal nerve, and sclerosing osteophytes was completely removed. A biopsy of the lesion showed sclerosing bone formation with thickened irregular trabeculae, which were infiltrated by reactive osteoblasts and chronic inflammatory cells (Fig. 2). This pathological feature confirmed previous diagnosis. No medications were given after the surgical procedure. Ten years after the operation, the patient is symptom-free with normal spinal motion. Radiography of the anteroposterior chest showed absence of any recurrent lesion (Fig. 3), and the patient did not have any further symptoms during the follow-up period.

## 3 Discussion

Chronic sclerosing osteomyelitis was first described by Garré in 1893 as a special form of osteomyelitis with characteristic thickening of and distended local bone.^[18,22]^ Patients with sclerosing osteomyelitis of Garré usually present with chronic pain and progressive swelling over a long period. However, this condition is usually not accompanied by suppuration or osteonecrosis.^[5]^ Recently, sclerosing osteomyelitis of Garré has been reported as a subtype of the synovitis, acne, pustulosis, hyperostosis, osteitis (SAPHO) syndrome in adults.^[2,23]^ For our patient, the clinical manifestation and laboratory examination results were nontypical. We did not detect any acne and pustule on his skin, the bacterial culture from puncture specimen was negative, and no lesion was found in his sternoclavicular joint. Therefore, it does not exactly conform to the characteristics of SAPHO syndrome.

The main differential diagnoses in this case were neoplastic diseases and inflammatory condition. Some malignant tumors such as osteosarcoma and Ewing sarcoma which are characterized by a rapidly progressive course have been ruled out according to clinical and radiographic features. Meanwhile, histological features of the present patient have obvious distinction with malignant tumors. These benign neoplasms such as osteoid osteoma and osteoblastoma have been considered. However, osteoid osteoma and osteoblastoma are relatively common osteogenic tumors with a distinct predilection for males between 10 and 20 years,^[24]^ which is not consistent with our case. In addition, there is no evidence to suggest that the patient has inflammatory condition. From the features of the clinical signs and radiography and biopsy of puncture specimen, the rib sclerosing osteomyelitis of Garré was initially diagnosed.

To date, extensive researches have been conducted on sclerosing osteomyelitis of Garré, which most commonly occurs among children and young adults (Table 1).^[20,21]^ Clinically, the chronic phase of the condition is always accompanied by recurrent pain and local swelling with an insidious onset.^[18]^ However, an undefined diagnosis remains the main issue. Researchers have reported that the Garré sclerosing osteomyelitis should involve a chronic disease course (typically, >3 months), evidence of chronic inflammation on biopsy, and lack of organism growth.^[16,25]^ For a definite diagnosis, the main criterion is analysis of the clinicoradiological features followed by a histological examination.

Conventional treatments have previously been attempted for the treatment of sclerosing osteomyelitis of Garré. High-dose antibiotics with NSAIDs have been reported to be effective.^[2,11,20]^ Some previous studies suggested that pamidronate could contribute to pain relief involved in this condition (Table 1).^[12,17,19]^ For our patient, surgical intervention was performed to completely alleviate the symptoms after the failure of conservative treatment. After a wide resection of the 10th intercostal nerve, sclerosing ribs and osteophytes, the pain of his left chest completely disappeared. The microbiological culture examination of the tissue near the sclerosing rib was negative, and antibiotics were not administered. The patient had recovered fully by follow-up at 10 years.

In summary, sclerosing osteomyelitis of Garré of the rib is an unusual condition and represents a noninfective course in the rib with a low morbidity. For the present patient diagnosed with rib sclerosing osteomyelitis of Garré, a wide resection of rib lesions without reconstruction obtained good results. However, further data and longer follow-up period are required to evaluate other therapies such as pamidronate and fenestration operation to arrive at a consensus regarding the diagnostic criteria and treatment strategies of rib sclerosing osteomyelitis of Garré. Through this case, we wish to report the condition of sclerosing osteomyelitis of Garré of the rib, which can be confused with malignant lesions, to highlight the existence of this rare entity.

## 4 Informed consent

The study was approved by the Institutional Review Boards of the Tianjin Medical University General Hospital, Tianjin, China. The patient signed informed consent for the publication of this case report and any associated images.
